# Exploring the Interplay Between Creative Self-Efficacy, Mindset, and Creativity in Response to Negative Feedback

**DOI:** 10.3390/bs15060806

**Published:** 2025-06-12

**Authors:** Mengrong Liu, Yilai Pei, Weiguo Pang

**Affiliations:** 1 School of Education, Zhejiang International Studies University, 299 Liuhe Road, Hangzhou 310023, China; 2 China Institute of Education and Social Development, Beijing Normal University, 19 Xinjiekouwai Street, Beijing 100875, China; peiyilai8128@gmail.com; 3 The School of Psychology and Cognitive Science, East China Normal University, 3663 North Zhongshan Road, Shanghai 200062, China

**Keywords:** negative feedback, creativity, creative self-efficacy, creative mindsets

## Abstract

Negative feedback is both common and important in the creative process. However, research has shown inconsistent effects of negative feedback on creativity, and individual characteristics may moderate the impact of negative feedback on creative performance. This study explores how creative self-efficacy and a creative developmental mindset interact to influence creative performance in response to negative feedback. One hundred and twenty-two university students were recruited to complete the Creative Self-Efficacy Scale and the Creative Mindset Scale. They also participated in two Alternative Uses Tasks and one Realistic Presented Problem, both before and after receiving pseudo negative feedback. The results indicated that individuals with high creative self-efficacy and a low developmental mindset generated more Realistic Presented Problem solutions after negative feedback, while those with high creative self-efficacy and a high developmental mindset generated more original Realistic Presented Problem solutions. These findings are discussed in relation to the self-enhancement and self-improvement motivations. Based on our results, the role of creative self-efficacy and a developmental mindset should be considered when delivering negative feedback. Our research also highlights ways to foster creativity in educational and workplace settings.

## 1. Introduction

### 1.1. Creativity and the Inconsistent Effect of Negative Feedback

Creativity is the process through which an individual’s or a group’s abilities interact with their environment to produce new and useful products in a social context (Plucker et al., 2004). Given that creativity involves the generation of novel and useful ideas, divergent thinking tests are widely recognized as effective measures of creativity (Runco & Acar, 2012). These tests often require participants to generate as many responses as possible that meet specific criteria. When assessing participants’ responses, evaluators consider both quantitative and qualitative aspects. Quantitative indicators focus on fluency, which measures the number of acceptable answers produced, while qualitative indicators emphasize originality, assessing whether the responses are novel (Acar et al., 2020).

Creativity is inherently social rather than isolated (Harrison & Dossinger, 2017), requiring evaluation within a contextualized audience. The 5A framework of creativity (Glăveanu, 2013) emphasizes five interdependent elements in creativity, namely, actor, action, artifact, audience, and affordance. “Audience” comprises collaborators, family members, opponents, colleagues, and the public who may accept, modify, or reject creative work. Similarly, the Systems Model of Creativity (Csikszentmihalyi, 1988, 1999) defines creativity as a phenomenon that emerges from the interaction of three interacting forces: the domain, which preserves and transmits validated ideas; the field, which consists of gatekeeping institutions that select which ideas merit recognition; and the individual, who both contributes novel ideas and can reshape the field itself. In this model, the field functions as the principal audience, determining which innovations endure. Without peer or expert evaluation, distinguishing creativity from mere absurdity is impossible. Thus, external feedback that reflects the audience’s appraisal is critical for both identifying and fostering creativity.

Feedback can be classified as positive or negative depending on whether the information signals that an individual’s performance meets a given standard (Kluger & DeNisi, 1996; Hattie & Timperley, 2007). While extensive research indicates that positive feedback typically enhances creativity (Lechermeier & Fassnacht, 2018), the impact of negative feedback on creative performance remains inconsistent (e.g., Dong et al., 2023; Geng et al., 2025; Li et al., 2024). On the one hand, negative feedback provides valuable insights to help creators identify the limitations of their work and the potential improvements it requires. These insights may prompt them to reduce the use of ineffective strategies, facilitate the formation of innovative solutions, and ultimately improve their creative performance (Y. J. Kim & Kim, 2020; Todd et al., 2022). On the other hand, negative feedback can potentially threaten creators’ self-worth, leading to negative emotions such as anger and frustration, which can impede their efforts and cause a decrease in creativity (Fong et al., 2018; Redifer et al., 2021). This research suggests that how individuals perceive and respond to negative feedback may have a significant impact on their creativity.

### 1.2. Creative Self-Efficacy

Negative feedback indicates that the individual’s current work does not meet creativity criteria, which can challenge their abilities to some extent. Thus, whether the individual still believes he or she is capable enough to accomplish the creative task, also known as creative self-efficacy (CSE; Tierney & Farmer, 2002), determines their subsequent response. Generally, individuals with high self-efficacy are more confident in achieving their goals and are also more likely to view negative feedback as an opportunity rather than a threat (Bandura, 2012; Bandura & Locke, 2003). They tend to adopt proactive coping strategies (S. Y. Lee et al., 2008) and typically achieve better performance (Judge & Bono, 2001).

Empirical research also supports the positive effects of CSE on creativity, especially in disadvantaged situations. Individuals with high CSE show greater originality in artistic tasks when negative feedback is anticipated or received (Hu et al., 2016). Employees with high CSE are more likely to engage in creative work in situations of job uncertainty than those with low CSE (Tang et al., 2020). CSE also positively affects the creativity of employees who are low in positive affect and high in negative affect (Thundiyil et al., 2016).

### 1.3. A Creative Mindset

The Mindset Theory (Dweck, 1999; Dweck & Leggett, 1988; Dweck & Yeager, 2019; Yeager & Dweck, 2020) proposes that when encountering challenges or setbacks, individuals’ inner beliefs about their ability shape their attributions, goal setting, and subsequent responses (Dweck, 2006). Broadly, mindsets fall into two negatively correlated categories: a developmental mindset views abilities as improvable through effort and learning, whereas a fixed mindset regards abilities as static and unchangeable. Karwowski (2014) has applied mindset theory to creativity, conceptualizing that individuals can have a creative developmental mindset (DM) or a fixed mindset (FM).

Since individuals with a high DM adopt performance goals that are focused on competence development and skill acquisition (Dweck, 1986), they are more likely to perceive negative feedback as nonthreatening and to invest greater effort in subsequent tasks (Zingoni & Byron, 2017). They set more ambitious goals, persist longer when confronted with failure, actively seek negative feedback, make more extensive revisions, and attain more creative outcomes (Cutumisu, 2019). Collectively, these findings underscore that a high DM fosters adaptive engagement and enhanced creativity in the face of negative feedback.

In contrast, individuals with an FM tend to prioritize performance goals (Y. Lee et al., 2022; A. A. Lee et al., 2024), focusing on outperforming others, showing off ability (performance-approach goals), and avoiding failure or being seen as incompetent (performance-avoidance goals). Since they view their efforts as evidence of incompetence, they are more likely to prove themselves or avoid challenges and withdraw in the face of negative feedback (e.g., Moser et al., 2011; Nussbaum & Dweck, 2008; Yeager et al., 2018).

Therefore, a high DM fosters adaptive engagement and creative growth, whereas a high FM elicits performance-approach or performance-avoidance strategies when confronted with negative feedback.

### 1.4. The Interaction Between Creative Self-Efficacy and a Creative Mindset

Integrating Bandura’s (2012) social cognitive theory with Dweck’s (2006) mindset theory, high CSE paired with a high DM engenders quality-focused responses to negative feedback. Confident in their capabilities, these individuals view challenges as surmountable and, guided by mastery goals, interpret negative feedback as informative rather than threatening. They allocate attention to task strategies and learning processes, leveraging negative feedback to refine and elaborate on their ideas (Kluger & DeNisi, 1996). Consequently, when both CSE and DM are high, individuals may generate more polished and qualified creative outcomes.

When confronted with negative feedback, individuals with an FM may adopt either performance-approach or performance-avoidance strategies, resulting in different outcomes. Crucially, the choice between these strategies is influenced by their self-efficacy: high self-efficacy predisposes individuals to performance-approach responses, whereas low self-efficacy favors performance-avoidance responses (Y. Lee et al., 2022).

Under a performance-approach orientation, individuals intensify efforts to demonstrate their competence and prove their abilities. Since they perceive negative feedback as a threat, they often attempt to “self-prove” by increasing the volume of their ideas. Conversely, performance-avoidance prompts individuals to withdraw from challenges and reduce their engagement so that they minimize the risk of exposing their perceived incompetence.

Importantly, negative feedback also reshapes one’s self-concept, which is an individual’s internal representation of their personal attributes and abilities (Markus & Wurf, 1987). Such feedback activates two key post-feedback motivations (Sedikides & Strube, 1997): self-improvement, which directs individuals to enhance their competence in light of new information and self-enhancement, which seeks to protect individuals’ positive self-view by demonstrating their existing ability.

In creative contexts, these motivations align with implicit theories of malleability: a DM fosters self-improvement, leading individuals to attribute setbacks to effort deficits and to engage in constructive, skill-building strategies (Karwowski, 2014; Dweck & Yeager, 2019). Conversely, an FM engenders self-enhancement, causing individuals to interpret failure as proof of incompetence and to adopt performance-approach or performance-avoidance strategies to defend their self-concept (Y. Lee et al., 2022; A. A. Lee et al., 2024). Empirically, self-improvement-oriented creators experience less threat and negative affect after negative feedback, employ remedial strategies, and sustain engagement. Self-enhancement-oriented individuals exhibit heightened distress, often producing a defensive surge of ideas or disengaging altogether (T.-Y. Kim et al., 2023; Hu et al., 2016; Baas et al., 2011).

### 1.5. The Present Study

Based on the above literature, this study aims to investigate how CSE and a creative mindset (DM vs. FM) interact to shape creative performance in the face of negative feedback. Specifically, this study aims to determine whether high CSE combined with a DM yields fewer but higher quality ideas and whether high CSE with an FM produces greater idea quantity but lower average quality.

Therefore, the following hypotheses were proposed:

**Hypothesis** **1:** *Under conditions of negative feedback and high CSE, a fixed mindset (low DM) will be associated with a greater quantity of creative ideas*.

**Hypothesis** **2:** *Under conditions of negative feedback and high CSE, a developmental mindset (high DM) will be associated with higher quality creative ideas*.

## 2. Methods

This study examined whether individuals’ creative developmental mindset moderates the relationship between creative self-efficacy and changes in creative performance following negative feedback.

### 2.1. Participants

G*Power (version 3.1) was used to calculate the required sample size for detecting a medium-sized interaction effect (f^2^ = 0.15) with an alpha level of 0.05, a power of 0.80, and three predictors, including the interaction term. Based on this, a minimum of 81 participants were needed. Participants were recruited from the subject pool of a university in eastern China. An online advertisement was posted on the institution’s research participation platform, and students volunteered to take part after providing informed consent. A total of 122 university students initially enrolled in this experiment and each received CNY 30 (~USD 4.20) for their participation. The study protocol was approved by the Human Subjects Protection Committee of the [blinded] university (approval number: HR517-2020, approval date: 5 October 2020).

To ensure the confidentiality and security of participant data, all responses were assigned unique anonymization codes, thereby dissociating individual identities from their data. Datasets were stored on password-protected, encrypted computers that were accessible only to the research team. Any publications derived from this study present data exclusively in anonymized form, with no personal identifying information disclosed.

Following data collection, five participants were removed. During a post-experiment debriefing in which they were asked to guess the study’s purpose, these participants explicitly indicated that they were aware that the negative feedback had been preprogrammed (e.g., stating that “the program deliberately gave me poor ratings” or “I’ve done similar experiments before, and I know that the negative feedback is definitely the experimental conditions”). After these exclusions, the final sample comprised 117 participants (27 males and 90 females; Mage = 20.33, SDage = 2.15).

### 2.2. Materials

**Creative self-efficacy** was measured using the Creative Self-Efficacy Scale (CSES) that was developed by Gong et al. (2009). The scale consists of four items (e.g., “I am confident in my ability to use creativity to solve problems”). Participants rated each item on a 5-point Likert scale, ranging from 1 (strongly disagree) to 5 (strongly agree). Higher scores indicate greater confidence in one’s ability to achieve creative outcomes. The scale demonstrated good reliability, with a Cronbach’s alpha of 0.85.

**Creative mindset** was measured by the Chinese version of the Creative Mindset Scale (CMS; Zhou et al., 2020), originally developed by Karwowski (2014). This scale consists of two sub-scales: the developmental mindset scale and the fixed mindset scale, each containing 5 items (e.g., “Everyone can create something great in some way if given the right conditions”). Participants responded on a 5-point Likert scale, from 1 (strongly disagree) to 5 (strongly agree). The Cronbach’s alpha for the developmental mindset was 0.83, while the fixed mindset was 0.57. Due to the low reliability of the fixed mindset scale, only the developmental mindset sub-scale was used for analysis.

**Creative performance** was measured by two types of tasks, the Alternative Uses Task (AUT) and the Represented Realistic Problem (RPP) task. Each task is evaluated based on fluency, originality, and flexibility metrics. The AUT required participants to list as many unconventional uses as possible for a specific common object (e.g., umbrella, sock) in two minutes. The RPP task required participants to generate as many novel and useful solutions as possible to a presented dilemma (e.g., “What do you do when your classmates are always interrupting you in class, making it impossible for you to keep up with the lessons”) within five minutes.

### 2.3. Calculation of Dependent Variables

An objective scoring method was adopted to evaluate the fluency, originality, and flexibility of the AUT and the RPP task (Runco, 2011; Runco & Albert, 1985).

**Fluency** refers to the number of answers provided by the participants, with each answer scored as 1 point. However, answers that describe the original uses of the object (e.g., umbrella—keeping out the rain) are not scored.

**Originality** measures the rarity of the answers. For each question, all participants’ responses were compiled into an answer pool. After merging similar responses, scores were assigned based on their proportion in the answer pool (≤1% scores 2 points, 1~5% scores 1 point, >5% scores 0 points). A participant’s originality score for a question is the sum of the originality scores for all their answers.

**Flexibility** refers to the number of categories represented in participants’ answers. All responses in the answer pool were classified into different categories, and the number of categories for a participant’s answers constituted the flexibility score. Given the subjectivity of classification, the snapshot method from Forthmann et al. (2018) was used. Two tasks were randomly selected and two raters independently classified the answers in the pool, with a consistency coefficient calculated between them. The results indicated good consistency in the flexibility classification for two AUTs (umbrellas ICC = 0.76, newspaper ICC = 0.89). The first author classified answers for other tasks.

Although pre-test and post-test tasks were randomly balanced, all indicators were standardized during data analysis to minimize the impact of differing task difficulties. The final dependent variables reflected changes in the creative indicators, calculated by subtracting the pre-test standardized scores from the post-test standardized scores.

### 2.4. Procedure

Participants read and signed the informed consent form, and then received unique identification numbers based on their registration order. They completed a questionnaire that included their number, age, sex, CSES, and CMS, followed by a creativity pre-test with 2 AUTs and 1 RPP task.

Next, participants were given a 5 min break while an anonymous evaluator reviewed their submissions and provided feedback. To increase the authenticity of the feedback, participants were prompted to enter their number to view their feedback. The feedback that was provided is stated below:

“*Participant x, based on your performance, the evaluator’s feedback is:*

*The creativity you demonstrated in these tasks is below average. The variety of uses you identified for the objects is limited and lacks originality. The solutions you proposed for realistic problems are not adequately innovative or practical*.”

After reviewing the feedback, participants completed the creativity post-test, which included 2 additional AUTs and 1 RPP. The order of the pre- and post-tests was randomized.

**Figure 1:**
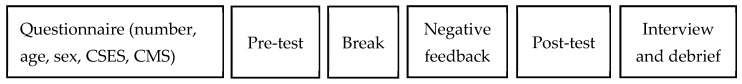
The procedure used in the experiment.

Upon finishing, they participated in a post-interview about their prior experience with similar tasks and their speculations about the study’s purpose. They were then informed of the true objective of the study and that the negative feedback was intentionally designed for the experimental conditions. They were also asked to keep the experiment’s details confidential to avoid influencing future participants. The entire experiment lasted about 25 min. Each participant received compensation for their participation (Figure 1).

### 2.5. Data Analysis

Data were analyzed using SPSS 24.0. First, descriptive statistics (means and standard deviations) and Pearson correlations among the primary variables were computed. Next, PROCESS 4.0 macro (Hayes, 2022) was employed with Model 1 specified, treating CSE as the predictor, each of the six creativity-change indices as the outcome, and developmental mindset as the moderator. Finally, for any significant interaction effects, simple slopes analyses were conducted at one standard deviation above, at, and below the mean of developmental mindset to probe the nature of the moderation and to determine how the CSE–creativity-change relationship differed under high, medium, and low developmental mindset conditions.

## 3. Results

Table 1 presents the descriptive and correlational results. There was no significant association between creative self-efficacy and a developmental mindset (*r* = 0.14, *p* = 0.14). Creative self-efficacy showed no significant associations with the dependent variables (i.e., changes in creativity indicators). A developmental mindset also had no significant associations with any of the other dependent variables, except for a weak positive correlation with changes in RPP originality (*r* = 0.19, *p* = 0.04). In addition, creative self-efficacy differed significantly by sex (*t*(115) = 3.40, *p* < 0.001), with males reporting higher levels of creative self-efficacy (M = 15.30, SD = 3.09, *n* = 28) than females (M = 13.10, SD = 2.80, *n* = 89).

**Figure 2:**
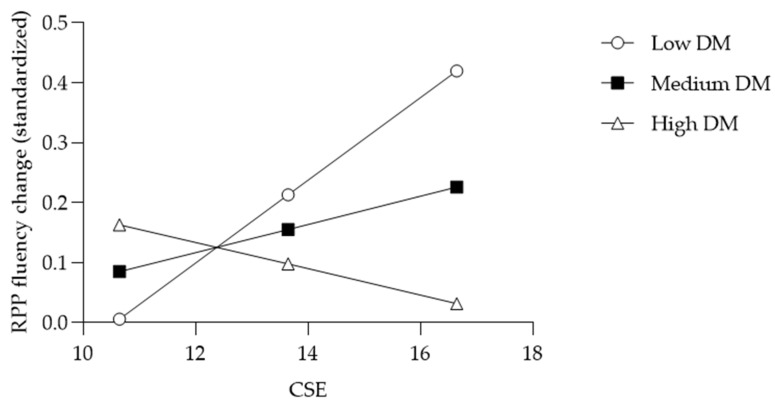
Conditional effect of CSE on RPP fluency change moderated by DM.

**Figure 3:**
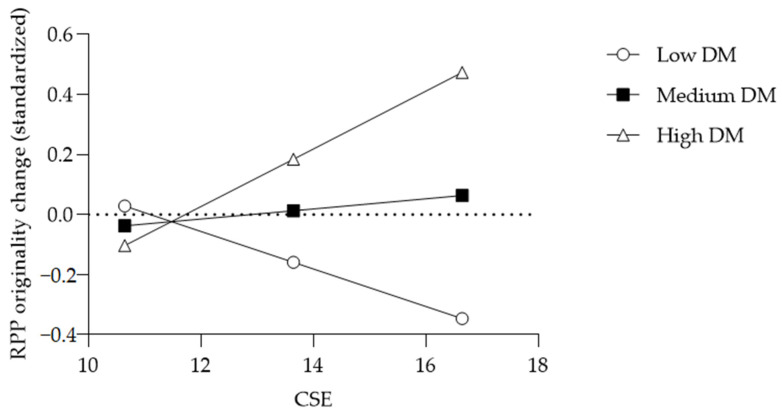
Conditional effect of CSE on RPP originality change moderated by DM.

To test the hypotheses, Model 1 of PROCESS 4.0 (Hayes, 2022) was used to examine the moderating effect of a developmental mindset on the link between creative self-efficacy and creativity changes following negative feedback. All variables were standardized to reduce multicollinearity and improve interpretability. The results demonstrated a significant interaction between creative self-efficacy and a developmental mindset on RPP fluency change (*B* = −0.14, *SE* = 0.06, *p* = 0.04) and RPP originality change (*B* = 0.24, *SE* = 0.09, *p* < 0.01). As shown in Table 2, Figure 2, and Figure 3, simple slopes tests suggested that RPP fluency change was only associated with increased creative self-efficacy in the low developmental mindset condition (*t* = 2.23, *p* = 0.03), and not in the medium and high developmental mindset conditions. RPP originality change was only marginally associated with increased creative self-efficacy in the high developmental mindset condition (*t* = 1.92, *p* = 0.06), and not in the medium and low developmental mindset conditions. Thus, H1 and H2 were mostly supported.

The results showed that the interaction between creative self-efficacy and a developmental mindset was not significantly associated with AUT fluency (*B* = 0.01, *SE* = 0.11, *p* = 0.93), AUT originality (*B* = 0.16, *SE* = 0.10, *p* = 0.13), AUT flexibility (*B* = −0.10, *SE* = 0.14, *p* = 0.51), or RPP flexibility (*B* = −0.07, *SE* = 0.09, *p* = 0.39).

## 4. Discussion

The ambivalent effects of negative feedback on creativity have increasingly attracted scholarly attention. While high CSE in individuals receiving negative feedback is generally viewed as beneficial, our study further hypothesized that a creative mindset would interact with CSE, leading to inconsistent effects on the quantity and quality of creativity.

### 4.1. Interaction Between CSE and DM on Creativity After Negative Feedback

Overall, our results support the hypothesis that CSE and mindset jointly shape divergent creative responses to negative feedback.

First, DM moderated the relationship between CSE and change in RPP fluency (*B* = −0.14, *SE* = 0.06, *p* = 0.04). Simple slopes analysis revealed that CSE predicted increased RPP fluency only among individuals with a low DM (*B* = 0.21, *SE* = 0.09, *p* = 0.03), but not among those with a medium or high DM. Thus, Hypothesis 1 was partly supported. In other words, participants who believed in their creative ability but viewed it as fixed generated more solutions in response to negative feedback. This pattern may reflect the dual influence of high CSE and a fixed mindset: CSE bolsters a positive self-concept (Bosson et al., 2003; Raskin et al., 1991), while a fixed mindset encourages maintenance of existing performance (Plaks & Stecher, 2007). Under these conditions, negative feedback threatens the self-concept (H. Y. Lee et al., 2019), activating self-enhancement motives that drive effort (Y. Lee et al., 2022). Heightened self-concept concern can also elicit strong negative arousal, which may facilitate idea fluency via a cognitive persistence pathway (De Dreu et al., 2008). Additionally, fixed-mindset individuals tend to process feedback superficially (Mangels et al., 2006), prioritizing quantity (i.e., number of responses) over depth to restore self-worth.

Second, DM moderated the relationship between CSE and change in RPP originality (*B* = 0.24, *SE* = 0.09, *p* < 0.01). Simple slopes analysis indicated that CSE predicted marginally greater originality only among individuals with a high DM (*B* = 0.29, *SE* = 0.15, *p* = 0.06), but not among those with a low or medium DM. Thus, Hypothesis 2 received partial support: participants who believed in both their creative ability and its malleability generated more original solutions following negative feedback. This may be because high CSE strengthens one’s self-concept and a developmental mindset frames negative feedback as a growth opportunity, thereby reducing negative affect (Hu et al., 2016) and activating self-improvement motivation. So, they can set higher goals, adopt adaptive coping strategies, and maintain strong beliefs in the efficacy of effort with fewer helpless attributions (Hong et al., 1999; Blackwell et al., 2007). Moreover, a developmental mindset directs attention toward goal-relevant information, promoting deep reflection and an emphasis on creative quality, especially originality (Mangels et al., 2006).

Taken together, our findings demonstrate that the benefits of high CSE under negative feedback are contingent on the DM and the specific creativity metric employed. High-CSE individuals with a low DM tend to exhibit greater idea fluency, whereas those with a high DM produce more original, higher quality solutions. This distinction refines prior work by Redifer et al. (2021) who observed that high CSE generally boosts creativity quantitatively under negative feedback, but that it does not account for mindset effects. Our results show that a low DM may drive only superficial quantity gains, whereas a high DM yields genuine qualitative improvements. Likewise, Tao et al. (2024) reported overall creativity gains with high CSE using a composite score, without differentiating quantity from quality. Furthermore, our findings echo those of Y. Lee et al. (2022) who showed that confident fixed-mindset individuals set ambitious performance goals in a reading task yet demonstrated poorer engagement and outcomes. Similarly, participants in our study with high CSE and a low DM mindset generated more ideas, but they did not outperform others on the fundamental creative criteria of originality.

### 4.2. Differences Between AUT and RPP Performance

In this study, no significant interaction between creative self-efficacy and a developmental mindset was found regarding the indicators of AUT and RPP flexibility. This may be attributed to the inherent differences in the AUT and RPP task. The AUT often lacks real-world context, focusing on abstract thinking about common objects. In contrast, the RPP task is closely tied to real-life scenarios, requiring participants to apply their creativity to practical issues. Furthermore, the RPP task is believed to necessitate both divergent and convergent thinking, reflecting different facets of creativity; however, the AUT primarily assess divergent thinking. Overall, the RPP task was more complex than the AUT, and individuals with high self-efficacy were more likely to choose challenging and engaging tasks (Tabernero & Wood, 2009). As a result, significant interactions were primarily evident in the RPP metrics.

### 4.3. Theoretical and Practical Implications

Our findings advance theory on feedback and creativity in two key ways. First, our results integrate Bandura’s social cognitive model and Dweck’s mindset framework, revealing that self-enhancement and self-improvement motives operate in tandem with CSE–mindset interactions to shape creative outcomes under negative feedback pressure. Second, the divergent patterns observed across fluency and originality metrics underscore the relevance of the dual evaluation framework of creativity, showing that high CSE can fuel either quantitative or qualitative gains depending on whether individuals have developmental or fixed mindsets.

For educators and creative mentors aiming to enhance performance, our findings suggest two integrated strategies. First, to foster higher quality outputs, curricula and mentorship programs should deliberately cultivate a developmental mindset while concurrently strengthening creative self-efficacy. Second, feedback should be strategically framed: presenting negative feedback as diagnostic guidance stimulates self-improvement motives in developmental-minded individuals, whereas minimizing evaluative threat cues (e.g., avoiding language that emphasizes ability deficits) can avert defensive, self-enhancement responses in those with fixed beliefs. By combining mindset development, self-efficacy enhancement, and solution-focused feedback, educators and mentors can effectively nurture both the quantity and quality of creative outputs across diverse learners and creators.

### 4.4. Limitations and Future Directions

This study has several limitations that warrant further research. First, our sample was drawn from a single university and exhibited a pronounced sex imbalance that yielded approximately three females for every male participant. Although sex was statistically controlled in the analysis, future research should recruit more balanced samples or conduct multisite studies to examine whether the observed CSE–mindset effects can be generalized across sexes and cultural contexts.

Second, the study employed bogus feedback in a laboratory setting to simulate negative feedback. Real-world negative feedback varies by source (e.g., peers, supervisors, clients) and delivery mode (e.g., written, verbal), all of which may differentially influence creative responses. Subsequent studies should manipulate the feedback source and format in field or quasi-field settings to assess ecological validity and boundary conditions.

Third, our cross-sectional design captures only a snapshot of CSE and mindset dynamics. Longitudinal and diary methods could chart how self-efficacy, mindsets, and creative performance co-evolve over time, revealing whether the interactions we observed are stable or transient.

Finally, qualitative or mixed-methods approaches could unpack the cognitive and affective processes that mediate the CSE–mindset interplay. By addressing these limitations, future research can deepen our understanding of how individual beliefs and environmental factors jointly shape creative resilience under evaluative pressure.

## 5. Conclusions

This study explored the moderating effect of the creative mindset on the relationship between CSE and creativity performance after negative feedback. Consistent with the hypotheses, the results suggest that a DM moderates the effects of CSE on creativity. For individuals with a low DM, high CSE prompts individuals to increase the number of responses. For individuals with a high DM, high CSE prompts individuals to increase response originality.

These findings extend existing theory by demonstrating that the benefits of CSE are not uniform but depend critically on one’s mindsets about ability, highlighting the importance of cultivating both CSE and a developmental mindset. By illuminating the dual-metric nature of creativity and the role of mindset as a boundary condition, our study provides a more nuanced blueprint for future research and practice. Further investigation of these interactive dynamics across varied tasks, feedback types, and populations will deepen our understanding of how individual beliefs shape creative resilience and performance when faced with different challenges.

## Figures and Tables

**Table 1:** Means, standard deviations, and correlation coefficients of all the variables (*n* = 117).

	Mean	SD	Min	Max	1	2	3	4	5	6	7	8	9	10	11	12	13	14	15	16	17	18	19	20	21
1 Sex	—	—	—	—	—																				
2 Age	20.31	2.11	18.00	27.00	−0.09	—																			
3 CSE	13.64	3.00	6.00	20.00	−0.30 ***	0.10	—																		
4 DM	18.15	2.77	9.00	25.00	0.00	−0.04	0.14	—																	
5 Pre-A-Flu	−0.11	1.76	−2.65	6.50	0.13	0.03	0.10	0.01	—																
6 Post-A-Flu	0.07	1.98	−3.57	5.74	0.19 **	0.03	0.17	−0.02	0.77 ***	—															
7 Pre-A-Ori	−0.19	1.73	−2.69	7.65	0.08	0.14	−0.01	0.05	0.34 ***	0.25 **	—														
8 Post-A-Ori	0.11	1.78	−3.37	6.86	0.07	0.09	−0.08	0.06	0.25 **	0.17	0.76 ***	—													
9 Pre-A-Fle	−0.01	1.67	−3.41	4.07	0.15	−0.02	0.08	−0.07	0.80 ***	0.67 ***	0.21 *	0.18	—												
10 Post-A-Fle	−0.03	1.83	−4.15	4.07	0.14	0.04	0.09	−0.09	0.64 ***	0.83 ***	0.10	0.13	0.54 ***	—											
11 Pre-R-Flu	−0.09	0.94	−1.47	2.78	0.11	0.02	0.15	−0.14	0.60 ***	0.52 ***	0.36 ***	0.23 *	0.51 ***	0.42 ***	—										
12 Post-R-Flu	0.04	0.93	−1.47	2.78	0.24 **	0.09	0.21 *	−0.22 *	0.47 ***	0.49 ***	0.15	0.01	0.43 ***	0.43 ***	0.63 ***	—									
13 Pre-R-Ori	−0.03	0.93	−1.40	3.25	0.04	0.06	−0.03	−0.21 *	−0.13	−0.11	−0.06	−0.03	−0.14	−0.07	−0.07	−0.08	—								
14 Post-R-Ori	0.01	1.00	−1.40	2.82	−0.05	0.12	0.01	−0.05	−0.03	0.02	0.22 *	0.30 ***	−0.00	0.03	−0.02	−0.02	0.34 ***	—							
15 Pre-R-Fle	0.00	1.01	−1.59	3.16	0.14	0.03	0.16	−0.09	0.55 ***	0.46 ***	0.32 ***	0.20 *	0.43 ***	0.35 ***	0.91 ***	0.56 ***	−0.07	0.02	—						
16 Post-R-Fle	−0.03	0.94	−1.59	2.48	0.19 *	0.19 *	0.09	−0.20 *	0.40 ***	0.39 ***	0.13	0.01	0.41 ***	0.33 ***	0.49 ***	0.78 ***	0.00	0.01	0.45 ***	—					
17 C-A-Flu	0.17	1.27	−2.89	3.95	0.12	0.00	0.13	−0.04	−0.18	0.49 ***	−0.08	−0.08	−0.07	0.41 ***	−0.02	0.12	0.01	0.06	−0.03	0.05	—				
18 C-A-Ori	0.30	1.21	−2.33	4.15	−0.00	−0.07	−0.10	0.02	−0.12	−0.11	−0.31 ***	0.38 ***	−0.04	0.05	−0.18 *	−0.19 *	0.04	0.13	−0.16	−0.18	0.00	—			
19 C-A-Fle	−0.02	1.68	−4.21	3.25	0.01	0.07	0.02	−0.03	−0.11	0.23 *	−0.10	−0.04	−0.41 ***	0.55 ***	−0.04	0.04	0.06	0.04	−0.05	−0.05	0.51 ***	0.08	—		
20 C-R-Flu	0.14	0.80	−2.14	2.11	0.16	0.07	0.06	−0.09	−0.18	−0.05	−0.26 **	−0.26 **	−0.11	−0.00	−0.44 ***	0.41 ***	−0.01	−0.01	−0.42 ***	0.33 ***	0.17	−0.02	0.11	—	
21 C-R-Ori	0.05	1.11	−3.44	3.12	−0.04	0.09	0.05	0.19 *	0.12	0.14	0.25 **	0.38 ***	0.13	0.12	0.08	0.07	−0.53 ***	0.59 ***	0.13	0.03	0.05	0.09	0.01	−0.02	—
22 C-R-Fle	−0.02	1.03	−3.23	2.22	0.04	0.17	−0.05	−0.08	−0.16	−0.09	−0.19 *	−0.19 *	−0.05	−0.03	−0.43 ***	0.18 *	0.07	−0.02	−0.55 ***	0.49 ***	0.08	−0.01	0.02	0.71 ***	−0.08

Notes: CSE = Creative self-efficacy; DM = Developmental mindset; Pre = Pre-test; Post = Post-test; A = Alternative Uses Task; R = Realistic Presented Problems; C = Change; Flu = Fluency; Ori = Originality; Fle = Flexibility. * *p* < 0.05, ** *p* < 0.01, *** *p* < 0.001.

**Table 2:** Conditional effects of DM and CSE on RPP fluency and originality change.

DM Conditions	*B*	*SE*	*t*	*p*	LLCI	ULCI
RPP fluency						
Low (−1 SD)	0.21	0.09	2.23	0.03	0.02	0.39
Medium (Median)	0.07	0.08	0.91	0.37	−0.08	0.22
High (+1 SD)	−0.07	0.11	−0.60	0.55	−0.28	0.15
RPP originality						
Low (−1 SD)	−0.19	0.13	−1.47	0.14	−0.44	0.07
Medium (Median)	0.05	0.11	0.47	0.64	−0.16	0.26
High (+1 SD)	0.29	0.15	1.92	0.06	−0.00	0.59

## Data Availability

Data will be made available upon reasonable request.

## Abbreviations

The following abbreviations are used in this manuscript:

CSE	Creative self-efficacy
CSES	Creative Self-Efficacy Scale
CMS	Creative Mindset Scale
DM	Developmental mindset
FM	Fixed mindset
AUT	Alternative Uses Task
RPP	Realistic Presented Problems
